# Facilitators and Barriers to Chronic Disease Self-Management and Mobile Health Interventions for People Living With Diabetes and Hypertension in Cambodia: Qualitative Study

**DOI:** 10.2196/13536

**Published:** 2020-04-24

**Authors:** Lesley Steinman, Hen Heang, Maurits van Pelt, Nicole Ide, Haixia Cui, Mayuree Rao, James LoGerfo, Annette Fitzpatrick

**Affiliations:** 1 Department of Health Services University of Washington Seattle, WA United States; 2 MoPoTsyo Patient Information Centre Phnom Penh Cambodia; 3 Division of General Internal Medicine University of Washington Seattle, WA United States; 4 General Medicine Service VA Puget Sound Health Care System Seattle, WA United States; 5 Department of Medicine University of Washington Seattle, WA United States; 6 Department of Global Health University of Washington Seattle, WA United States; 7 Departments of Family Medicine University of Washington Seattle, WA United States; 8 Department of Epidemiology University of Washington Seattle, WA United States

**Keywords:** diabetes mellitus, hypertension, chronic disease, noncommunicable diseases, health educators, mHealth, qualitative, disease management, developing countries

## Abstract

**Background:**

In many low- and middle-income countries (LMICs), heart disease and stroke are the leading causes of death as cardiovascular risk factors such as diabetes and hypertension rapidly increase. The Cambodian nongovernmental organization, MoPoTsyo, trains local residents with diabetes to be peer educators (PEs) to deliver chronic disease self-management training and medications to 14,000 people with hypertension and/or diabetes in Cambodia. We collaborated with MoPoTsyo to develop a mobile-based messaging intervention (mobile health; mHealth) to link MoPoTsyo’s database, PEs, pharmacies, clinics, and people living with diabetes and/or hypertension to improve adherence to evidence-based treatment guidelines.

**Objective:**

This study aimed to understand the facilitators and barriers to chronic disease management and the acceptability, appropriateness, and feasibility of mHealth to support chronic disease management and strengthen community-clinical linkages to existing services.

**Methods:**

We conducted an exploratory qualitative study using semistructured interviews and focus groups with PEs and people living with diabetes and/or hypertension. Interviews were recorded and conducted in Khmer script, transcribed and translated into the English language, and uploaded into Atlas.ti for analysis. We used a thematic analysis to identify key facilitators and barriers to disease management and opportunities for mHealth content and format. The information-motivation-behavioral model was used to guide data collection, analysis, and message development.

**Results:**

We conducted six focus groups (N=59) and 11 interviews in one urban municipality and five rural operating districts from three provinces in October 2016. PE network participants desired mHealth to address barriers to chronic disease management through reminders about medications, laboratory tests and doctor’s consultations, education on how to incorporate self-management into their daily lives, and support for obstacles to disease management. Participants preferred mobile-based voice messages to arrive at dinnertime for improved phone access and family support. They desired voice messages over texts to communicate trust and increase accessibility for persons with limited literacy, vision, and smartphone access. PEs shared similar views and perceived mHealth as acceptable and feasible for supporting their work. We developed 34 educational, supportive, and reminder mHealth messages based on these findings.

**Conclusions:**

These mHealth messages are currently being tested in a cluster randomized controlled trial (#1R21TW010160) to improve diabetes and hypertension control in Cambodia. This study has implications for practice and policies in Cambodia and other LMICs and low-resource US settings that are working to engage PEs and build community-clinical linkages to facilitate chronic disease management.

## Introduction

### Background

There is an increasing burden of noncommunicable diseases (NCDs) across the world and particularly in low- and middle-income countries (LMICs) as they go through the epidemiological transition from infectious to chronic diseases that accompanies economic development [1]. In 2016, NCDs accounted for 60% of disability-adjusted life years (DALYs) lost, a common measure of disease burden, and for 71% of all deaths [2]. Most deaths from cardiovascular diseases now occur in LMICs [3], and 80% of global deaths in LMICs are from NCDs [4].

Cambodia is experiencing the same epidemiological transition as other LMICs. Life expectancy is on the rise, increasing from an average of 56 and 60 years in 1990 to 66 and 72 years in 2015 for men and women, respectively [5]. The 2016 Global Burden of Disease Study’s visualizations show an increasing burden of NCDs, with NCD DALYs increasing by 55% from 1990 to 2016 for countries such as Cambodia with a low-to-middle sociodemographic index [6]. In Cambodia, diabetes, stroke, and heart disease have demonstrated the largest percentage change in years lived with disability since 1990. Ischemic heart disease has been the leading cause of death since 2005, and cerebrovascular disease became the second leading cause of death in 2015 [5]. A large proportion of deaths due to heart disease, stroke, and diabetes are attributable to metabolic risks such as high blood pressure, cholesterol, glucose, and BMI, all of which are modifiable risk factors that can be managed or prevented outside of the health system.

The 2010 World Health Organization’s (WHO) Stepwise Approach to Surveillance survey first found a prevalence of diabetes and hypertension of 2.9% and 11.2%, respectively, in the general Cambodian population [7], but this had rapidly increased to 9.6% and 14.2% in 2016, respectively, when that survey was repeated. These prevalence rates are higher in more vulnerable populations such as among Cambodians living with HIV [8]. Although a variety of methods have been developed to assist patients in managing their diabetes or hypertension and to increase access to care, the use of community- and peer-based interventions seems especially promising for reaching underserved populations [9-11]. In a review by Joshi et al [12] on studies involving task shifting for the management of chronic conditions in LMICs, it was found that nonphysician health workers could successfully screen patients and improve patients’ control of blood pressure and glucose levels when compared with usual health care. Lay health workers also have more time to provide behavioral counseling services and patient education as clinicians in LMICs often have limited time to spend with patients [13-17].

Mobile health (mHealth) offers a promising approach for managing chronic conditions in LMICs. mHealth is a subset of electronic health and encompasses a wide range of mobile and wireless technologies to help improve health and health care [18]. mHealth typically supports four functions: (1) health promotion and awareness, (2) remote monitoring and care support, (3) disease surveillance and outbreak detection, and (4) decision support system [19,20]. mHealth interventions can provide opportunities for improved chronic disease management in LMICs, given the limited health system infrastructure [21] and high cell phone coverage (90%) [22,23]. In Cambodia, 94% of the people report owning their own phone, with 99% being reachable by phone [24]. mHealth can also support people living with chronic conditions outside of the health care system where they spend most of their time and can benefit from regular self-management and routine long-term monitoring [25-27].

Although it is promising, more evidence is needed for mHealth for the management of chronic conditions in LMICs. Most mHealth evidence to date is on communicable diseases (eg, tuberculosis) and on maternal and child health [28]. A recent 2016 systematic review found eight randomized controlled trials (RCTs) to date of mHealth interventions to support chronic conditions in LMICs [19], specifically in China, Honduras, India, Malaysia, Mexico, Pakistan, Taiwan, and Uruguay. The mHealth interventions included two studies on health promotion, five studies on remote monitoring and care (eg, appointment reminders and medication adjustments), and one study on a clinical decision support system. The studies targeted people living with diabetes, hypertension, and asthma, and all studies but one reported some improvements in their outcomes of interest, which included clinical measures, symptoms, health service utilization, and self-rated health. As such a diversity of interventions, outcomes, and diseases was targeted by these mHealth interventions, it is hard to generalize these findings.

### Objectives

We aimed to contribute to the research and practice about chronic disease management in Cambodia by conducting an exploratory study to identify the facilitators and barriers to diabetes and hypertension management and user preferences for mHealth-based messages for chronic disease self-management and community-clinical linkages.

Our specific formative research questions were as follows:

1. What are people living with diabetes and/or hypertension currently doing to manage their chronic condition?

2. What are the facilitators and barriers to managing diabetes and/or hypertension, from the perspectives of people living with diabetes and/or hypertension (patients) including peer educators (PEs)?

3. What are the user (patient and PE) preferences for the format and content of cell phone messages to support people living with diabetes and/or hypertension, including PEs?

4. What are the facilitators and barriers to using mHealth technology from the perspectives of people living with diabetes and/or hypertension, including PEs?

We used these findings to develop an mHealth mobile-based messaging intervention to improve chronic disease management in Cambodia. As such, our fifth research question was as follows:

5. How acceptable, appropriate, and feasible are the mHealth messages to people living with diabetes and/or hypertension, and what revisions are needed to improve the mHealth messages before the RCT effectiveness-implementation study?

This qualitative study is the first phase of a larger RCT to test the effectiveness and implementation of the mHealth intervention to improve clinical outcomes via better evidence-based disease management practices (eg, self-management, regular monitoring of blood pressure and glucose, and medication adherence) for people living with diabetes and hypertension in Cambodia.

## Methods

### Design

We used an exploratory qualitative study design to better understand patient and PE perspectives on chronic disease management and mHealth. We used the 32-item checklist Consolidated Criteria for Reporting Qualitative Research [29] to guide the design, data collection, analysis, and reporting of our research study. We received institutional review board approval from the University of Washington Human Subjects Division and the Cambodian National Ethics Committee for Health Research.

### Theoretical Framework

**Figure 1:**
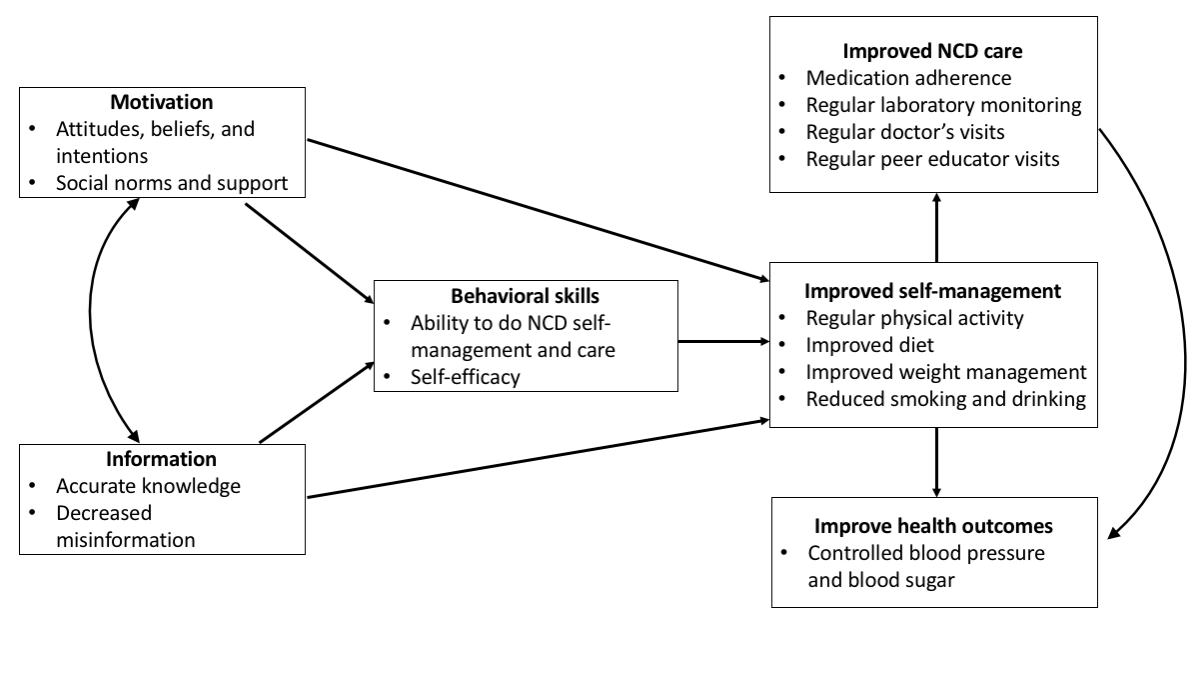
Modified information-motivation-behavioral model for improving diabetes and hypertension management through a mobile health intervention in Cambodia. NCD: noncommunicable disease.

We used the information-motivation-behavioral (IMB) theoretical framework [30] to guide data collection and mHealth message development. The IMB is a well-established behavior change model that has been applied to other diabetes self-management interventions to improve clinical and care outcomes [31,32]. Figure 1 shows our modified IMB model for this study. The IMB suggests three main determinants of starting and maintaining health behaviors: (1) accurate information (which includes reducing misinformation) that can be easily translated into health behavior changes, (2) personal and social motivation to act on this information, and (3) behavioral skills to implement the health behavior with confidence and effectiveness [30]. Our study will gather information on facilitators and barriers to chronic disease management to craft mHealth messages that can improve knowledge, motivation, and skills for self-management. Improving these three behavioral determinants can then improve chronic disease care (medication adherence, regular monitoring of blood pressure and glucose, regular doctor consultations, and regular visits with PEs) and ultimately clinical outcomes (controlled blood pressure and blood sugar). In addition, improved health outcomes can in turn motivate people to maintain change, which may reduce intervention fatigue and improve the sustainability of the intervention.

### Participants and Setting

The study was conducted in close partnership with the Cambodian nongovernmental organization (NGO), MoPoTsyo Patient Information Centre [33-35]. MoPoTsyo was established in 2005 to improve NCD management in Cambodia using the six strategies for improving the quality of chronic disease care, as recommended by the chronic care model [36]: self-management support, decision support, delivery system design, clinical information systems, health care organization, and community resources. MoPoTsyo trains people living with diabetes and/or hypertension to serve as PEs for their community, similar to a lay health worker or community health worker model. These PEs provide education and support for chronic conditions (eg, nutrition guidelines and self-monitoring of blood glucose and pressure) as well as linkages with the health system for regular laboratory profiles, medical consultations, and routine medication dispensing. To facilitate community-clinical linkages and chronic disease management, MoPoTsyo maintains a centralized database that tracks participants’ demographics and health outcomes, laboratory results, doctor’s visits, and pharmacy invoices when patients pick up medications.

### Recruitment

We recruited the study participants from two poor urban areas in the capital, Phnom Penh, and four areas where PE networks cover rural operational districts: Chamkar Leu, Baray-Santuk, Kampong Speu, and Kong Pisey. These six areas were purposefully selected using maximum variation sampling [37] to represent different patient demographics and access to MoPoTsyo’s chronic disease management services. We used convenience sampling to select focus group (FG) and interview (IW) participants from these six areas. For FGs with people living with diabetes and/or hypertension (called *patients* by MoPoTsyo), the eligibility criteria included active participation in MoPoTsyo’s peer education program and being present for their doctors’ consultation on the day of the FG. PEs notified patients of the FG opportunity and explained that they were not required to participate to receive other services from MoPoTsyo. For the IWs, all PEs were invited to participate from these six geographical areas. We obtained written informed consent from both patients and PEs.

### Data Collection

The study team developed a semistructured IW and FG guide to better understand patient and PE perspectives on chronic disease management and mHealth. The guide included main questions and follow-up questions and probes depending on how participants answered the main questions. Participants could skip any question that they did not wish to answer. We also conducted a brief demographic survey at the start of each FG and IW. Data were collected by trained qualitative researchers: HH conducted the FGs and half of the IWs in Khmer and LS conducted half of the IWs in the English language with a Khmer interpreter. The FGs lasted 90 min and the IWs lasted 20 to 30 min. Each group included 8 to 12 participants with a similar composition of mixed ages and genders. Refreshments were provided at each group and IW, and the participants were provided a small amount of cash to reimburse them for the personal cost of their time and transportation. The FGs and IWs took place at the community health center or a nearby restaurant where participants came regularly and were familiar with.

### Data Analysis

Hard copies and electronic data were stored securely and without any identifiable information. We analyzed the survey data using Stata 14 [38] for descriptive statistics. FGs and IWs were audio recorded and transcribed, and the recordings were destroyed one month following transcription. We analyzed the transcripts using a deductive thematic analysis, using Atlas.ti for data management. A thematic analysis is a method for identifying, analyzing, organizing, describing, and reporting themes found within a dataset [39]. It applies a well-structured approach to understand and organize key features of the data and for highlighting the similarities and differences across diverse research participants and for generating unanticipated insights [40]. We used the six phases for the thematic analysis articulated by Nowell et al [40]: familiarizing ourselves with the data; coding data using a standardized coding scheme [41,42]; searching for themes from the coded and collated text; reviewing coded text within each theme to add additional codes or themes and collapse themes with insufficient or inconsistent data; defining, naming, and describing the scope and content of each theme and revising after a research team review; and producing a summary report of the findings to provide an account of the data across and within themes and quotes to illustrate each theme. We used this report to create mobile phone voice messages for the chronic disease management mHealth intervention.

We used Lincoln and Guba’s [43] criteria for credibility, transferability, dependability, and confirmability to help establish the trustworthiness of our data. For credibility, or goodness of fit between participants’ perspectives and how we represented these perspectives, we used prolonged engagement with the data, research triangulation with multiple members of our team, and data collection triangulation with the IW and FG data. For transferability, we provided descriptions of the setting, participants, and their perspectives so that the readers can determine whether the findings are applicable to their context. For dependability and confirmability, we created an audit trail of codes and themes to document decisions made throughout the course of the study.

### Mobile Health Message Development

We used findings from IWs and FGs to develop mHealth cell phone messages to improve MoPoTsyo patients’ clinical outcomes (eg, blood pressure and glucose). Specifically, the messages were created to improve evidence-based chronic disease management via better access to health care (doctor’s consultations, laboratory monitoring, medications, and PEs) and self-management (diet, weight management, physical activity, alcohol, and smoking). We created a matrix with each of these eight categories and grouped current practices and facilitators and barriers to disease management by each of these categories. For each of these categories, we then developed message content aimed at improving knowledge, motivation, and skills to either strengthen the facilitators or address barriers that were identified in the FGs. We used the findings about how mHealth would help or hinder disease management to inform the format of the messages (eg, frequency and duration of messages). The research team reviewed the draft messages together to come up with final messages to record and pilot test.

We pilot tested four messages with 5 MoPoTsyo patients to assess message acceptability, appropriateness, and feasibility using open-ended discussion questions and a brief multiple-choice survey. In the survey, patients were asked to rate on a 5-point Likert scale (1=strongly disagree to 5=strongly agree) whether the messages: got their attention, were believable, were convincing, and were important to them; whether the messages put thoughts in their mind and helped them feel able to change how they were managing their chronic disease; and whether they agreed with or enjoyed the messages. We used input from the discussions and from the ratings to then revise the messages using this feedback before the RCT began.

## Results

### Participants

The study engaged 70 participants: 59 individuals (*patients*) and 11 PEs living with diabetes, hypertension, or both diabetes and hypertension (Table 1). Patients had a mean age of 55.9 years (SD 9.1), were 63% (37/70) women, completed on average 4.2 years (SD 3.2) of education, had smoked in the last 30 days (9/59, 15%), and had been in MoPoTsyo’s program for approximately 3 years (SD 2.82). A majority of these patients were living with both diabetes and hypertension (43/59, 73%), and over 70% (33/46) with diabetes and 75% (42/56) with hypertension) of them felt they were successfully managing their disease. A greater proportion of PEs were men (6/11, 55%), were educated (mean years of school 8.6, SD 4.3), felt they were successfully managing their chronic conditions (over 10/11, 90% with diabetes and 8/8, 100% with hypertension), and enrolled in MoPoTsyo’s program (mean 4.9 years, SD 2.3).

A summary of findings on patients’ (FG) and PEs’ (IW) perspectives on chronic disease management and mHealth support is provided in the following sections, along with illustrative quotes from patients and from PEs living with diabetes, hypertension, or both diabetes and hypertension.

**Table 1:** Study participants (N=70).

Participant characteristics		MoPoTsyo patients (focus groups)	Peer educators (interviews)	Total
Number of participants, n		59	11	70
Age (years), mean (SD)		55.9 (9.1)	52.8 (6.5)	55.4 (8.8)
**Sex, n (%)**				
	Female	37 (63)	5 (45)	42 (60)
	Male	22 (37)	6 (55)	28 (40)
**Education**				
	School completed (years), mean (SD)	4.20 (3.15)	8.55 (4.25)	4.89 (3.67)
DM^a^+HTN^b^, n (%)		43 (73)	8 (73)	51 (72)
DM only, n (%)		3 (5)	3 (27)	6 (9)
HTN only, n (%)		13 (22)	0 (0)	13 (19)
**Successfully managing DM?, n (%)**				
	Yes, or more yes than no	33 (72)	10 (91)	43 (75)
	No, or more no than yes	13 (28)	1 (9)	14 (25)
**Successfully managing HTN?, n (%)**				
	Yes, or more yes than no	42 (75)	8 (100)	50 (78)
	No, or more no than yes	14 (25)	0 (0)	14 (22)
Smoked in the past 30 days, n (%)		9 (15)	2 (18)	11 (16)
Length of time in the peer educator program (years), mean (SD)		2.9 (2.8)	4.9 (2.3)	3.2 (2.8)

^a^DM: diabetes.

^b^HTN: hypertension.

### Chronic Disease Management: Knowledge, Attitudes, and Practices

Both patients and PEs were well informed about the recommended diabetes and hypertension management practices. Participants were knowledgeable about the following evidence-based practices: (1) taking daily medications to manage their blood pressure and blood glucose; (2) eating less salt and more fruits and vegetables; (3) exercising or other physical activities; (4) regularly meeting with their PE to receive support and education regarding their self-management activities; to monitor diabetes and hypertension outcomes; to discuss symptoms and complications; to discuss their medical prescription; to monitor their blood pressure, blood glucose, and weight; and to discuss their yearly visits for laboratory and doctor consultations. As one participant shared:

We need to do physical exercise and follow a healthy diet to reduce blood glucose and so on. I also take medicines regularly.

FG3

Very few participants mentioned traditional remedies for chronic disease management.

Although individuals and PEs knew about evidence-based chronic disease management strategies, there were deficits in IMB skills to carry out these strategies on a regular basis. Knowledge gaps included misunderstandings about what constitutes the appropriate frequency, intensity, and duration of physical activity and how to incorporate dietary changes into their lifestyle. As one patient shared:

For me, the most significant content is about physical exercise—how to do physical exercise properly and what are the advantages of doing physical exercise?

FG6

Challenges to incorporating recommended dietary changes into their daily routine included that household meals are prepared by other household members and that other household members prefer more salt and sugar for better taste, they work long days and get fatigued when they do not eat their typical foods, and healthier foods are more expensive and harder to access.

MoPoTsyo helps with access to care by providing services as close as possible to where people live and having a fixed low price for medications, laboratory tests, and consultations that patients get through their PE network membership. That said, even with this assistance from MoPoTsyo, resources such as time and money are limited for accessing care as many people live in rural areas and spend half a day travelling to and from and waiting for services as there is a very limited public transportation system in Cambodia. As one PE shared:

At the start of the month, their children get salary for working and they have money to come hospital, so there are more patients. But at the end of the month, there are fewer and fewer patients to come because they do not have money for firstly, transportation cost, secondly, consultation fee and thirdly, medicine payment. For the furthest village from Orm Laing, they have to spend about 6 to 7.50 USD for transportation, this doesn’t include expense for food.

IW9

Access to quality care is further limited in rural areas where doctors have varying training in chronic disease management and laboratory monitoring is not offered regularly. Patients may also forget to take medicines regularly or may have difficulty taking medicines with food when they are working during the day. As one patient described:

My problem is that I am mostly absent-minded. For instance, I am rushing to go to work in the morning! I do not have time to have breakfast, so I plan to have breakfast at workplace, and then I forget to take my medicines.

FG4

Family and peer support are important cultural norms that facilitate chronic disease management, as well as having routines and feeling better when they follow recommended strategies. For example:

We ask their family to help remind them about consultation appointment; especially the patients with hypertension to see their doctor regarding the appointment because they may not feel badly.

IW9

Every morning, even when I was very busy, I prepared my medicines on my desk. So, if I walked around in my house, I took a look to see my medicines on my desk, then I recognized immediately, and I took the medicines soon. So I never miss in taking the medicines.

FG4

### Mobile Health for Chronic Disease Management

Patients and PEs viewed mHealth mobile-based messages as an acceptable, feasible, and appropriate intervention to support chronic disease management. Cell phones are increasingly accessible in Cambodia—one FG participant shared that “Nowadays, Cambodian people are having a mobile phone in every house and a person can have more than one phone” [FG3]. However, participants highlighted that older, poorer, and rural communities have less access or have less phone literacy even if they have access to a phone. mHealth messages are seen not just as ways to educate, support, and remind patients but also as a way to support PEs who can in turn reinforce the messages. Most participants preferred voice messages over text messages, given the small screen size on older phones, limited literacy and low vision (eg, a participant from FG1 shared that she would need her child to read text messages for her), and a preference for a familiar, friendly voice from a trusted organization (MoPoTsyo). Suggestions included “announcing MoPoTsyo before the messages start so they will trust the messages” [IW4] and “having a rhythm of a diabetes song so when I hear the song, I know it’s MoPoTsyo!” [FG4]. A few PEs preferred text messages so that they could share them more easily with their patients.

Limited interactivity was desired (“just pressing the answer key and listening” [FG3]) given the low education and digital literacy of many middle-aged and older Cambodians. Participants recommended that messages be simple, interesting, and brief—as one participant requested, “use daily words; do not used technical words! Since we are farmers, please use simple words, then we can understand, if you use the words that we never heard before, it will be difficult to understand!” [FG5]. It was desired that messages be sent two times per week during dinnertime so that patients know when to expect them, could be available (not working or out on errands), have access to their cell phones (since many share phones with spouses or children), and have their family around to support them in listening to and carrying out message recommendations. As one PE described:

two times per week is enough; if we send the messages more often, it will be hard and it could annoy their time; the patients would feel boring with the messages and then that would be a problem; or the patients may not have to time for messages.

IW11

Participants also wanted to be able to listen to the mHealth messages more than once and did not see each message as a onetime resource or as an individual-level intervention—they saw utility for their whole family and community for promoting health.

Sharing phones with family members was seen as both a facilitator and barrier to using an mHealth intervention for chronic disease management. As described earlier, sharing phones meant that family members would often be around when they received the mHealth messages, providing support, reinforcement, and reminders about what was shared. However, sharing phones meant that the targeted MoPoTsyo participant was not always near the phone through which the message was sent. One PE shared:

The difficulty is that the person who handles the phone is not always staying near the patients; the phone is with their children who are working far from home, while their parents who are patients are staying at home! So the message may not be received by the patients but by their children; even though their children will convey the message content, it is not like what the patients will hear the message by them directly!

IW8

Other barriers to cell phone access include cell phone numbers changing often for patients to get low promotional SIM cards and limited cell phone literacy. As one participant shared:

My husband has one, my child has one, but I don’t have one—I don’t know how to use the phone!

FG6

In addition, participants viewed mHealth as an intervention that could help connect the dots for chronic disease management, supplementing the information, motivation, and skills they receive from their PE as well as linking them to needed medicines, doctor consultations, and laboratory monitoring. One PE shared:

The messages through a mobile phone can be an additional support to educate patients besides the verbal explanation by peer educators to patients. The messages could help improve patient’s practice lifestyle such as attitude in taking medicines, physical exercise and checking blood pressure and blood glucose! The message is a reminder or a doctor to remind patient to practice a healthy lifestyle. [IW9])

However, they did not see mHealth as a panacea as it cannot fully solve problems for patients having limited time, money, and other resources to manage their conditions. As one patient described:

I don’t have any difficulties [using the phone], but on the day I have to come to buy medicines, I don’t have much time because I need to look after my grandchild at home. It is hard for me.

FG1

### Mobile Health Message Development

Table 2 shows the four messages that were pilot tested. During the pilot test, patients strongly agreed with the message’s acceptability, agreeability, and feasibility, with a 80%-100% (4/5-5/5) rating of the survey statements as *strongly agree*. Furthermore, the advisory committee suggested shortening and simplifying several of the messages that were too long or complex and changing the voice to a more upbeat, jovial female voice from the more formal, masculine voice that was pilot tested.

Overall, 34 mHealth messages were created to improve chronic disease management for Cambodians living with diabetes, hypertension, or both conditions. Guided by the IMB model, the messages were (1) *informational* (eg, reminders that it was time to pick up medications, get laboratory tests, or go to their doctor’s consultations and education about what constitutes healthy food); (2) *motivational* (eg, rationales for why exercising, diet, and medications are important for chronic disease management and supportive messages that acknowledge how hard it can be to manage their chronic condition, such as eating less salt when not in charge of cooking for the family), and (3) *behavioral* (eg, creative strategies for incorporating regular medications, physical activity, and healthy eating into their daily life). Multimedia Appendix 1 provides information on each of the mHealth messages, including the message script, length of message (in seconds), type (IMB framework), chronic disease management topic, and target (whether the message was sent to all MoPoTsyo patients or a subset of MoPoTsyo patients).

In the RCT effectiveness study of the mHealth messaging intervention [44], the messages will be targeted and tailored to MoPoTsyo participants according to clinical and service outcomes. For example, patients due to pick up their medications will receive the following message:


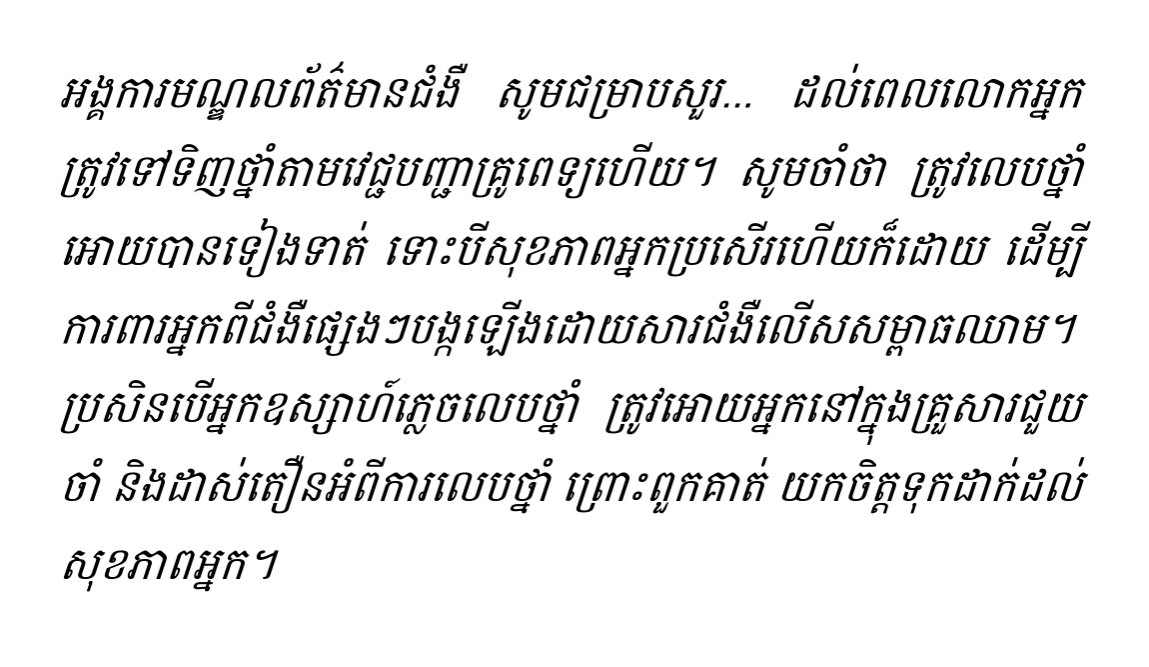

It is time to go to the pharmacy with your patient-book to buy your medicine for your prescription [Information and Behavior]. Remember—taking your medicine every day even if you do not feel sick will help prevent serious complications. [Information, Motivation, and Behavior] If you sometimes forget to take medicine, ask your family to help you remember when to take your medicine [Behavior]. They care about you!

Motivation

**Table 2:** Messages for pilot testing.

Message ID	Khmer script	English script
A1	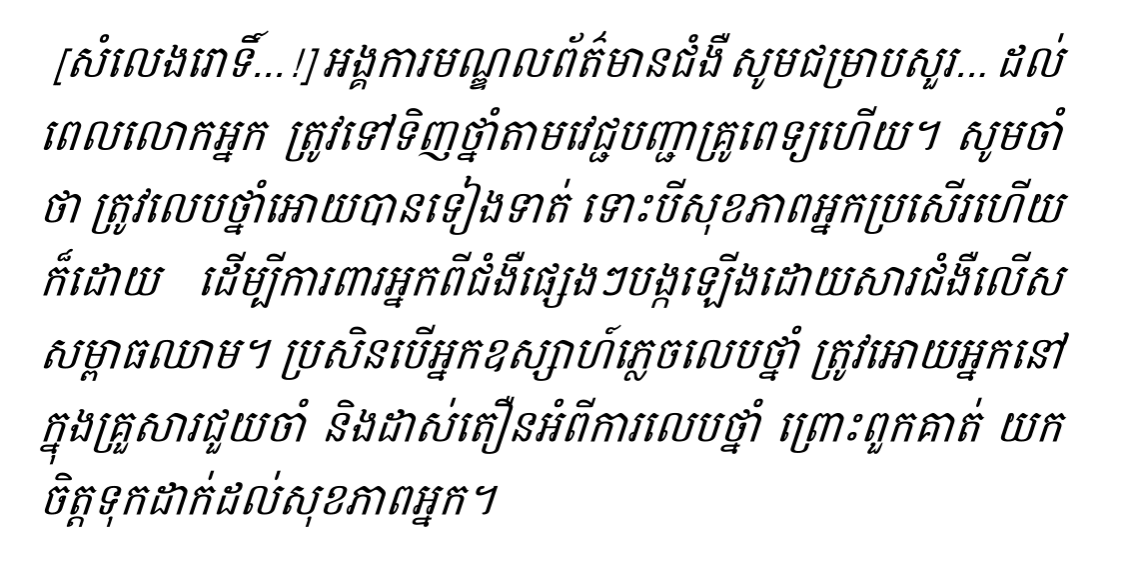	It is time to go to the pharmacy with your patient-book to buy your medicine for your prescription. Remember—taking your medicine every day even if you do not feel sick will help prevent serious complications. If you sometimes forget to take medicine, ask your family to help you remember when to take your medicine. They care about you!
B3	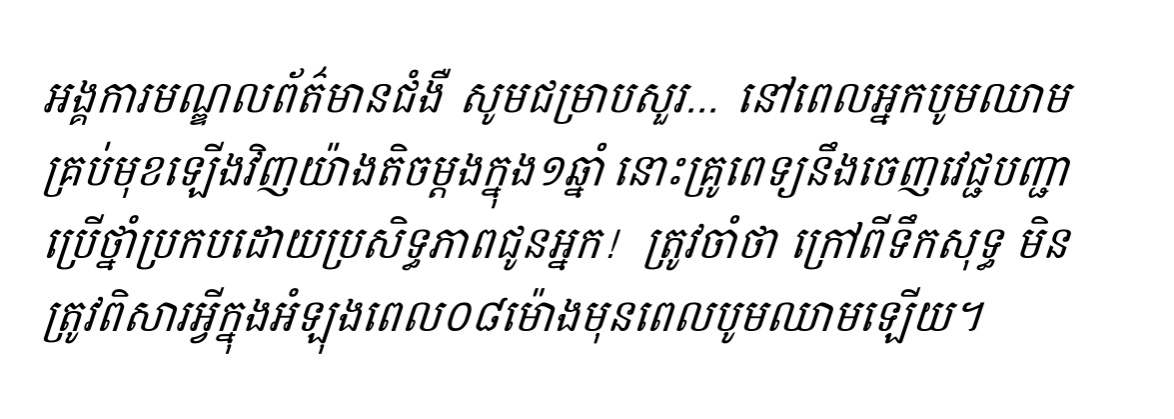	By updating the lab profile at least one time per year the Doctor can see if he is prescribing the right treatment specially for you. Remember to not eat or drink anything except pure water for 8 hours before the blood draw.
C2	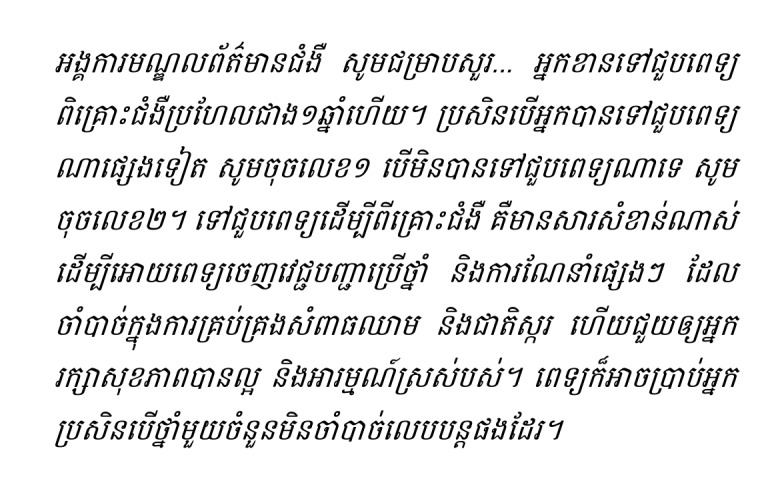	It looks like you did not see the doctor for more than 1 year. If you went to another doctor for your diabetes or high blood pressure, press #1, if not, press #2. Going to your doctor is important so that they can prescribe the medications and other things you need for your blood pressure and blood sugar, to stay healthy and feel better. They also can tell you whether there are any medications that you do not need to take any more.
D1	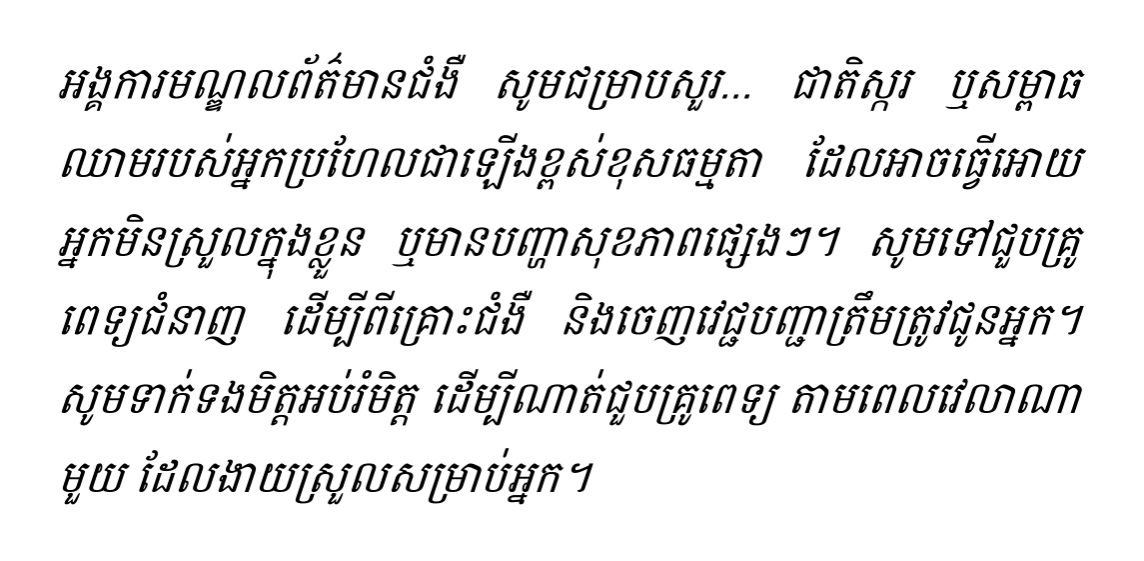	It looks like your blood sugar (blood pressure) is too high which may make you feel worse or have other health problems. Please go to see a Doctor who can prescribe the right medicines for you. You can also contact your PE to get an appointment with a doctor.

## Discussion

### Summary of Findings

In this study, we identified the facilitators and barriers to diabetes and hypertension management in Cambodia from the perspectives of people living with these chronic conditions, including trained lay health workers (PEs) to support chronic disease management. Not surprisingly, given their involvement in the PE network, many people living with chronic conditions are aware of the best practices for disease management (eg, medications, regular doctor’s visits and laboratory monitoring, physical activity and healthy eating, and less smoking and alcohol use). However, challenges remain on how to best incorporate these practices into daily lives. Key barriers include limited time and resources to access medications, clinical support, and recommended guidelines for physical activity (eg, aerobic and muscle strengthening activities) and healthier diets with reduced sodium intake. Access to effective, quality chronic care remains to be a challenge, particularly in rural areas.

This study also identified the preferred mHealth mobile-based message format and content to inform our RCT of an mHealth intervention to strengthen self-management for diabetes and hypertension and to improve community-clinical linkages to existing services and support systems in Cambodia. Study participants see mHealth as providing opportunities for reminders about medications, laboratory studies, and doctor visits; education about how to include best practices for chronic disease management where they live and work; and support for those barriers that cannot easily be overcome. Many participants have access to cell phones and prefer simple, short voice messages over texting because of limitations in literacy, vision, and cell phone facilities as well as the trust conveyed by a familiar voice. Participants also prefer that messages be delivered at dinnertime to increase the likelihood that they will receive the messages and can engage their family and friends for support in their disease management. We did not find any major differences in perspectives on chronic disease management and mHealth between people living with chronic conditions (FG discussions) and those persons who were trained as PEs (one-on-one IWs).

### Comparison With Existing Literature

The literature on chronic disease management in Cambodia is scarce. The few existing peer-reviewed publications highlight a variety of limitations and challenges in screening and diagnosis, service delivery, financing and insurance schemes, health workforce, drug supply, health information systems, and governance [45,46]. These findings are similar to many other studies conducted in LMICs specific to the management of hypertension and diabetes. Other common health service challenges reported in these studies include an insufficient number of trained health care workers coupled with a high patient load, few diagnostic capabilities especially for glycated hemoglobin, low remuneration of health workers often leading to low motivation, no electronic databases for monitoring patient data, limited services available in rural or community settings, and few trained specialists such as ophthalmologists or podiatrists [13,14,16,17,47-50]. Poor access to essential medicines is also reported as a common challenge in LMICs, where the drug stock is often unavailable, particularly in government subsidized facilities as well as rural settings [13,16,51,52]. These challenges in health service delivery prompted the creation of MoPoTsyo in 2005 to empower patients by (1) training and maintaining a lay health workforce that can support chronic disease self-management and (2) creating a financially sustainable system for continuous access to low-cost medications, laboratory monitoring, and doctor consultations that cooperates with Cambodia’s public health care system.

As reported in our study, the financial burden for ongoing management of chronic conditions such as hypertension and diabetes are one of the most typical barriers that patients face. Financial barriers can include the cost of clinical care (eg, doctor fees, laboratory tests, medications, and catastrophic health expenditures), higher costs for healthy food, and transport costs for traveling to health facilities or pharmacies [13,14,16,17,47,49,53]. Khatib et al [54] reported that in rural LMICs, expenditures on hypertension medications alone can constitute up to 49% (IQR 20-100) of a household’s capacity to pay. Some patients are forced to borrow or take money from friends or relatives, spend less on other household needs, or are unable to renew their required prescriptions in full or on time [50,55]. Although the costs of clinical care and medications within the MoPoTsyo network are more affordable than health services outside the network, participants still reported a cost burden from medications, laboratory tests, and annual visits with a physician. These costs were both direct monetary costs for these services as well as indirect costs from transportation or spending the day to travel for clinical care. This suggests additional structural support and system changes are needed to supplement an mHealth intervention and the access to care provided via MoPoTsyo’s negotiated low, fixed prices for health services.

Patients also face a variety of personal challenges in implementing changes to their behaviors and lifestyle, as was confirmed by our study. A variety of sources in LMICs report that the most common self-management practices for people living with diabetes and hypertension are medication use, diet control, and physical activity [55-59]. The literature discussing patient-reported barriers to medication adherence describes many similar challenges reported by participants in our study, including unwanted side effects, dislike of taking medications, the belief that medications are not necessary, and financial and access barriers, as described in the previous paragraph [16,55,58]. Adherence to a recommended diet is also a common patient-reported barrier, both in the literature from LMICs and in the results from our study. Challenges include understanding how to implement diet changes [57]; ease of access to unhealthy foods, particularly foods high in fat, salt, and sugar [17,58]; problems obtaining healthy food at home if they are not the primary cook in the household [49,58]; and still feeling hungry after eating the recommended diet [55]. Other patient barriers reported in the literature include cultural norms that are not conducive to healthy behaviors [48], not having access to devices for regular self-monitoring of blood glucose or blood pressure [49,60], and a preference of traditional remedies over Western medications [16,17]. These barriers were not cited frequently by either MoPoTsyo participants or the PEs.

Social support emerged as a key factor for both chronic disease management and for using mHealth to support disease management. The MoPoTsyo PE network [33] is built on the evidence that trained members of the patient community can provide social support (along with education, identification, and referral) for people living with chronic conditions [61]. Family members have often been identified as the main source of social support available to people living with diabetes and hypertension in LMICs [62], yet this can be both a facilitator and barrier, as was identified in this study. Specifically, although family members can provide instrumental support (eg, driving patients to appointments) and emotional support (eg, helping patients cope with their disease), recommended lifestyle changes can conflict with regular family routines and family members may provide misinformation or increase the stress levels of patients [63]. Future mHealth interventions may benefit from engaging family members more directly given their key role in both cell phone access and usability and from facilitating recommended disease management strategies around diet and exercise and obtaining medications and accessing health care providers.

As described earlier, it is too early to conclude whether mHealth messaging interventions for chronic conditions in developing countries are effective or not [19], with studies showing mixed results [64-71]. Although it is not exactly clear what contributes to the success of mobile messaging programs, it is likely that features such as the specific content, frequency, timing, personalization, interactive capabilities, and usability can affect outcomes. For example, most interventions with successful outcomes reported sending participants messages at least once a week, whereas daily messages were typically viewed as intrusive by participants and sending messages less than once a week was too infrequent to have an impact. Similarly, according to the opinions of participants in our study, most preferred to receive two messages per week.

From the available literature, it appears that most mHealth messaging programs utilized only text messaging; few incorporated voice messages into the intervention [66,72,73]. However, our results showed that participants often preferred voice over text messages because of low literacy (both limited ability to read and to use technology), poor vision, limited familiarity with sending and receiving text messages, higher trust in a familiar voice, and the ability to replay messages if desired. These findings are not unique in low-income settings; for example, a study in India found that 89% of participants preferred receiving voice reminders for their medications over text messages [74]. Even with voice messages, more targeted and tailored dissemination efforts may be needed to reach older and poorer patients with less cell phone access and lower technological literacy.

### Strengths and Limitations

The primary strength of this study is our conducting of formative research with our target audience to identify the facilitators and barriers to chronic disease management to develop an mHealth messaging intervention for improving self-management and access to care. We worked in close partnership with a successful Cambodian NGO (MoPoTsyo) to design an mHealth intervention for a real-world setting and patient population. Our MoPoTsyo coauthors were involved in each phase of the study and received training in research methods to build the capacity for future research studies. The lead author (LS) has over 15 years of experience conducting community-engaged, cross-cultural, qualitative research with patients and providers. The second and third authors (HH and MP) lead MoPoTsyo’s work including engagement, training, monitoring, and evaluation. For the past 15 years, the last two authors (JL and AF) have worked closely with MoPoTsyo and separately with the lead author on health promotion research and practice with low-resource settings in the United States. This was the lead author’s first project with MoPoTsyo and in Cambodia; as such, partnering with the study coauthors and the MoPoTsyo team was imperative to yield relevant findings to improve NCD outcomes in Cambodia. Over the course of the study, we also consulted with an advisory committee of key stakeholders (see Acknowledgements) to ensure the messages were designed for dissemination and sustainability and were aligning with national policies and priorities such as the *National Strategic Plan for Prevention and Control of Noncommunicable Diseases 2013-2020*.

The limitations of the study are common to formative research studies. We used a convenience sample of participants in an existing PE network; as such, our findings may not be generalizable to other contexts. FGs as a data collection method may also foster group thinking in which only some perspectives are shared. In this study, the participants knew each other from participating together in MoPoTsyo’s peer networks, which may have influenced what responses were shared. In addition, although the use of the IMB framework is well established for designing individual behavior change interventions, newer models such as Opoku et al’s mHealth realist framework [75] can be useful for evaluating the implementation of the mHealth intervention to provide a more systematic appraisal of contextual factors that support or hinder intervention implementation, sustainability, and scale-up [76]. Their realist framework combines two models to articulate mechanisms between individual and contextual factors and access to care: Anderson’s Behavioral Model of Health Services Utilization [77] calls out three key factors that determine access to care: predisposing characteristics (eg, age and beliefs), enabling resources (eg, availability of providers), and need (eg, disease burden). Davis’s Technology Acceptance Model [78] adds perceived usefulness and perceived ease of use as two additional factors that lead to use and acceptance of technology.

### Conclusions

This formative study adds to the growing literature on using mHealth to support the self-management of diabetes and hypertension in LMICs. It provides a model for developing culturally appropriate mHealth NCD interventions in LMICs that are based on patient-identified factors. Specifically, this study identified the facilitators and barriers to chronic disease management and to using an mHealth messaging intervention to improve disease self-management and community-clinical linkages in a PE network in the Cambodian context. Our formative research informed the creation of 34 IMB mHealth messages that are currently being tested for effectiveness and implementation in a cluster RCT of 75 health center coverage areas in Cambodia. Our findings align with the existing literature on the facilitators and barriers to chronic disease management and mHealth messaging interventions in LMICs, suggesting that there may be implications for other settings. This study will help build the needed evidence for successful mHealth messaging interventions for NCDs in LMICs.

## Appendix

Multimedia Appendix 1 mHealth message script, length, information-motivation-behavioral content, chronic disease management topic, and target audience (N=34).

## Abbreviations

DALY: disability-adjusted life year
FG: focus group
IMB: information-motivation-behavioral
IW: interview
LMIC: low- and middle-income country
mHealth: mobile health
MOH: Ministry of Health
NCD: noncommunicable disease
NGO: nongovernmental organization
RCT: randomized controlled trial
WHO: World Health Organization
